# Supplementary figures and images for: Brain vascular stability relies on PAK2–cilia–PDGF-BB–HSPGs on basolateral side of endothelium
Source: Life Sci Alliance. 2026 Jan 5;9(3):e202503460. doi: 10.26508/lsa.202503460 (PMC12769094; doi:10.26508/lsa.202503460)

**PDGF-BB**

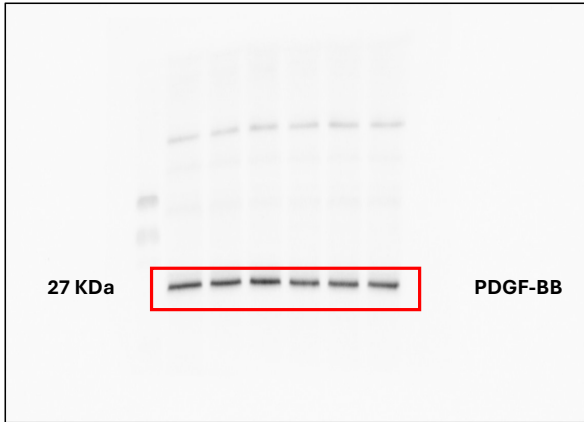

**ARL13B**

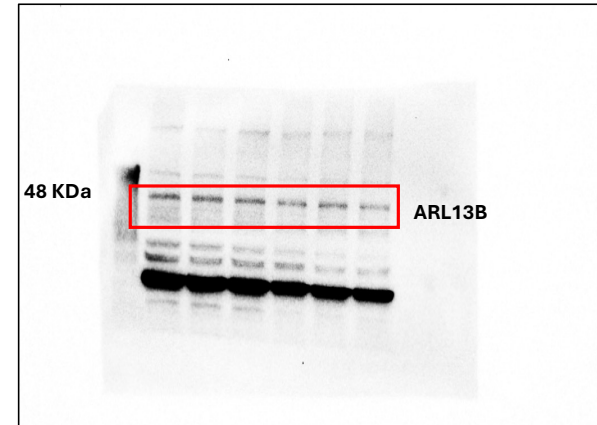

**PAK2**

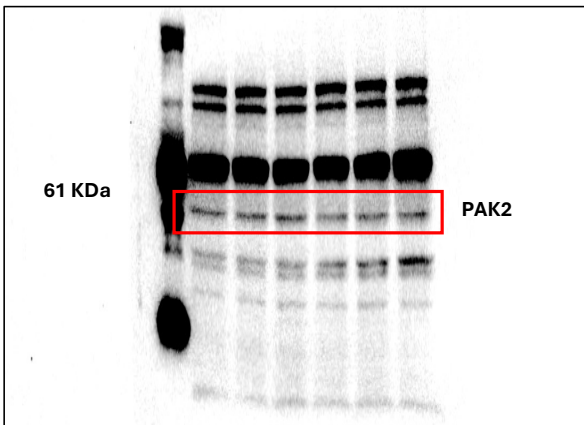

**$\beta$ -actin**

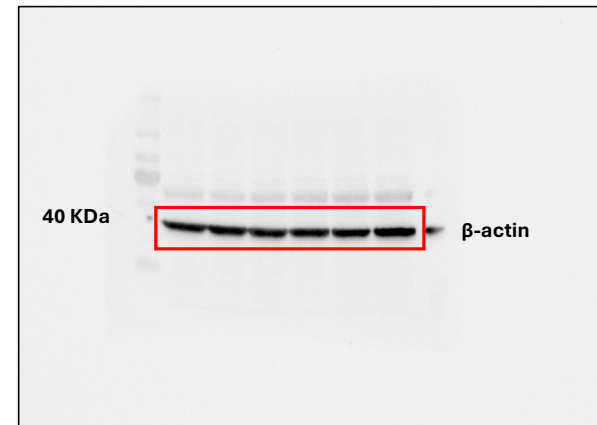

Supplement: Supplementary file 1 [file LSA-2025-03460_SdataF6.pdf]
